# Supplementary material for: Point-of-care diagnostic tests for influenza in the emergency department: A cost-effectiveness analysis in a high-risk population from a Canadian perspective
Source: PLoS One. 2020 Nov 16;15(11):e0242255. doi: 10.1371/journal.pone.0242255 (PMC7668582; doi:10.1371/journal.pone.0242255)
Supplement: S2 File — (PDF) [file pone.0242255.s002.pdf]

S2 File. Calculations used for treatment appropriateness

Patients with influenza

"Appropriate (tx – flu)"= $\frac{\text{(number of patients with influenza treated)}}{\text{(total number of patients with influenza)}}$

"Inappropriate tx (no tx – flu)" = $\frac{\text{(number of patients with influenza not treated)}}{\text{(total number of patients with influenza)}}$

Patients without influenza

"Appropriate (no tx – no flu)" = $\frac{\text{(number of patients not treated, did not have influenza)}}{\text{(total number of patients without influenza)}}$

"Inappropriate (tx – no flu)" = $\frac{\text{(number of patients treated, did not have influenza)}}{\text{(total number of patients without influenza)}}$

Notes:

"Inappropriate (no tx – flu)" and "Appropriate (tx - flu)" are complementary and sum up to 1.

"Appropriate (no tx – no flu)" and "Inappropriate (tx – no flu)" are complementary and sum up to 1.
