# Supplementary material for: Point-of-care diagnostic tests for influenza in the emergency department: A cost-effectiveness analysis in a high-risk population from a Canadian perspective
Source: PLoS One. 2020 Nov 16;15(11):e0242255. doi: 10.1371/journal.pone.0242255 (PMC7668582; doi:10.1371/journal.pone.0242255)
Supplement: S1 Table — (PDF) [file pone.0242255.s003.pdf]

S1 Table. Sensitivity analyses results

Parameter: Probability of pretest ILI being influenza (pInfluenza)
Range: 0 to 1

pInfluenza	Strategy	Cost (CAD)	Effectiveness	NHB (QALYs)
0	Treat Everyone	509.94	15.27745	15.26725
0	Batch PCR - Treat	536.0656	15.27813	15.26741
0	Batch PCR - Wait	528.097	15.27826	15.2677
0	NAAT	508.7696	15.27828	15.26811
0	DIA	489.2722	15.27827	15.26849
0	RIDT	488.1884	15.2783	15.26853
0	Clinical Judgement	477.2268	15.27811	15.26857
0	Don't Treat Anyone	468	15.2783	15.26894

pInfluenza	Strategy	Cost (CAD)	Effectiveness	NHB (QALYs)
1	Don't Treat Anyone	1441.575	13.31875	13.28992
1	Clinical Judgement	1406.358	13.4477	13.41957
1	RIDT	1421.069	13.46706	13.43864
1	Batch PCR - Wait	1453.108	13.51357	13.4845
1	DIA	1390.101	13.58045	13.55265
1	NAAT	1397.53	13.62648	13.59853
1	Batch PCR - Treat	1404.964	13.65903	13.63094
1	Treat Everyone	1343.751	13.67694	13.65006

CAD, Canadian dollars; DIA, digital immunoassay; NAAT, nucleic acid amplification test; PCR, polymerase chain reaction; QALY, quality-adjusted life year; RIDT, rapid influenza diagnostic test

Parameter: Probability of treatment within 48 hours of symptom onset (pTx48)
Range: 0.36 to 0.60

pTx48	Strategy	Cost (CAD)	Effectiveness	NHB (QALYs)
0.36	Don't Treat Anyone	608.1947	14.99613	14.98396
0.36	Clinical Judgement	612.2985	15.01054	14.99829
0.36	RIDT	623.9918	15.01288	15.0004
0.36	Batch PCR - Wait	662.9833	15.01887	15.00561
0.36	DIA	621.5829	15.02568	15.01324
0.36	NAAT	639.7982	15.03089	15.01809
0.36	Batch PCR - Treat	664.5564	15.03443	15.02114
0.36	Treat Everyone	633.5555	15.03588	15.0232

pTx48	Strategy	Cost (CAD)	Effectiveness	NHB (QALYs)
0.6	Don't Treat Anyone	608.1947	14.99613	14.98396
0.6	Clinical Judgement	609.7689	15.01846	15.00626
0.6	RIDT	621.0824	15.02199	15.00957
0.6	Batch PCR - Wait	659.6457	15.02932	15.01612
0.6	DIA	616.4491	15.04175	15.02942
0.6	NAAT	633.7614	15.04978	15.03711
0.6	Batch PCR - Treat	657.8811	15.05533	15.04217
0.6	Treat Everyone	626.5289	15.05787	15.04534

CAD, Canadian dollars; DIA, digital immunoassay; NAAT, nucleic acid amplification test; PCR, polymerase chain reaction; QALY, quality-adjusted life year; RIDT, rapid influenza diagnostic test

Parameter: Probability of death (non-ICU hospitalized) after early treatment (pDie_NonICU_Early)
Range: 0.06 – 0.10

pDie_NonICU_Early	Strategy	Cost (CAD)	Effectiveness	NHB (QALYs)
0.06	Don't Treat Anyone	608.1947	14.99613	14.98396
0.06	Clinical Judgement	611.0217	15.02239	15.01017
0.06	RIDT	622.5233	15.02652	15.01407
0.06	Batch PCR - Wait	661.2986	15.03451	15.02129
0.06	DIA	618.9916	15.04974	15.03736
0.06	NAAT	636.7511	15.05918	15.04645
0.06	Batch PCR - Treat	661.187	15.06572	15.0525
0.06	Treat Everyone	630.0087	15.06881	15.05621

pDie_NonICU_Early	Strategy	Cost (CAD)	Effectiveness	NHB (QALYs)
0.1	Don't Treat Anyone	608.1947	14.99613	14.98396
0.1	Clinical Judgement	611.0217	15.00733	14.99511
0.1	RIDT	622.5233	15.0092	14.99675
0.1	Batch PCR - Wait	661.2986	15.01464	15.00142
0.1	DIA	618.9916	15.01918	15.0068
0.1	NAAT	636.7511	15.02324	15.01051
0.1	Batch PCR - Treat	661.187	15.02598	15.01276
0.1	Treat Everyone	630.0087	15.02698	15.01438

CAD, Canadian dollars; DIA, digital immunoassay; NAAT, nucleic acid amplification test; PCR, polymerase chain reaction; QALY, quality-adjusted life year; RIDT, rapid influenza diagnostic test

Parameter: Probability of death (ICU hospitalized) after early treatment (pDie_ICU_Early)
Range: 0.21 – 0.35

pDie_ICU_Early	Strategy	Cost (CAD)	Effectiveness	NHB (QALYs)
0.21	Don't Treat Anyone	608.1947	14.99613	14.98396
0.21	Clinical Judgement	611.0217	15.01484	15.00262
0.21	RIDT	622.5233	15.01783	15.00538
0.21	Batch PCR - Wait	661.2986	15.02455	15.01132
0.21	DIA	618.9916	15.03441	15.02203
0.21	NAAT	636.7511	15.04115	15.02842
0.21	Batch PCR - Treat	661.187	15.04579	15.03256
0.21	Treat Everyone	630.0087	15.04783	15.03523

pDie_ICU_Early	Strategy	Cost (CAD)	Effectiveness	NHB (QALYs)
0.35	Don't Treat Anyone	608.1947	14.99613	14.98396
0.35	Clinical Judgement	611.0217	15.01419	15.00197
0.35	RIDT	622.5233	15.01708	15.00463
0.35	Batch PCR - Wait	661.2986	15.02369	15.01046
0.35	DIA	618.9916	15.03309	15.02071
0.35	NAAT	636.7511	15.03961	15.02687
0.35	Batch PCR - Treat	661.187	15.04408	15.03085
0.35	Treat Everyone	630.0087	15.04603	15.03343

CAD, Canadian dollars; DIA, digital immunoassay; NAAT, nucleic acid amplification test; PCR, polymerase chain reaction; QALY, quality-adjusted life year; RIDT, rapid influenza diagnostic test

Parameter: Probability of adverse event from treatment (pAE)
Range: 0.056 – 0.094

pAE	Strategy	Cost (CAD)	Effectiveness	NHB (QALYs)
0.056	Don't Treat Anyone	608.1947	14.99613	14.98396
0.056	Clinical Judgement	611.0217	15.01458	15.00236
0.056	RIDT	622.5233	15.01749	15.00504
0.056	Batch PCR - Wait	661.2986	15.02418	15.01095
0.056	DIA	618.9916	15.03381	15.02143
0.056	NAAT	636.7511	15.04045	15.02772
0.056	Batch PCR - Treat	661.187	15.04504	15.03182
0.056	Treat Everyone	630.0087	15.04719	15.03459

pAE	Strategy	Cost (CAD)	Effectiveness	NHB (QALYs)
0.094	Don't Treat Anyone	608.1947	14.99613	14.98396
0.094	Clinical Judgement	611.0217	15.01448	15.00226
0.094	RIDT	622.5233	15.01747	15.00502
0.094	Batch PCR - Wait	661.2986	15.02411	15.01088
0.094	DIA	618.9916	15.03376	15.02138
0.094	NAAT	636.7511	15.0404	15.02766
0.094	Batch PCR - Treat	661.187	15.04492	15.03169
0.094	Treat Everyone	630.0087	15.04677	15.03417

CAD, Canadian dollars; DIA, digital immunoassay; NAAT, nucleic acid amplification test; PCR, polymerase chain reaction; QALY, quality-adjusted life year; RIDT, rapid influenza diagnostic test

Parameter: Disutility of adverse event (uAE)
Range: 0.012 – 0.20

uAE	Strategy	Cost (CAD)	Effectiveness	NHB (QALYs)
0.012	Don't Treat Anyone	608.1947	14.99612	14.98396
0.012	Clinical Judgement	611.0217	15.01452	15.0023
0.012	RIDT	622.5233	15.01747	15.00502
0.012	Batch PCR - Wait	661.2986	15.02413	15.01091
0.012	DIA	618.9916	15.03378	15.0214
0.012	NAAT	636.7511	15.04042	15.02768
0.012	Batch PCR - Treat	661.187	15.04497	15.03174
0.012	Treat Everyone	630.0087	15.04693	15.03433

uAE	Strategy	Cost (CAD)	Effectiveness	NHB (QALYs)
0.02	Don't Treat Anyone	608.1947	14.9961	14.98393
0.02	Clinical Judgement	611.0217	15.01436	15.00214
0.02	RIDT	622.5233	15.01742	15.00497
0.02	Batch PCR – Wait	661.2986	15.02403	15.01081
0.02	DIA	618.9916	15.0337	15.02132
0.02	NAAT	636.7511	15.04034	15.0276
0.02	Batch PCR - Treat	661.187	15.04479	15.03156
0.02	Treat Everyone	630.0087	15.04634	15.03374

CAD, Canadian dollars; DIA, digital immunoassay; NAAT, nucleic acid amplification test; PCR, polymerase chain reaction; QALY, quality-adjusted life year; RIDT, rapid influenza diagnostic test

Parameter: Cost of oseltamivir (cTx)
Range: $34 - $42

cTx	Strategy	Cost (CAD)	Effectiveness	NHB (QALYs)
34	Don't Treat Anyone	607.0514	14.99613	14.98398
34	Clinical Judgement	608.3831	15.01453	15.00237
34	RIDT	621.3494	15.01748	15.00505
34	Batch PCR - Wait	659.8154	15.02414	15.01095
34	DIA	617.642	15.03379	15.02144
34	NAAT	635.483	15.04043	15.02772
34	Batch PCR - Treat	658.4582	15.04498	15.03181
34	Treat Everyone	622.0687	15.04698	15.03454

cTx	Strategy	Cost (CAD)	Effectiveness	NHB (QALYs)
42	Don't Treat Anyone	608.2034	14.99613	14.98396
42	Clinical Judgement	611.0416	15.01453	15.00231
42	RIDT	622.5321	15.01748	15.00503
42	Batch PCR - Wait	661.3098	15.02414	15.01092
42	DIA	619.0018	15.03379	15.02141
42	NAAT	636.7607	15.04043	15.02769
42	Batch PCR - Treat	661.2076	15.04498	15.03176
42	Treat Everyone	630.0687	15.04698	15.03438

CAD, Canadian dollars; DIA, digital immunoassay; NAAT, nucleic acid amplification test; PCR, polymerase chain reaction; QALY, quality-adjusted life year; RIDT, rapid influenza diagnostic test

Parameter: Cost of Batch PCR test (cBPCR)
Range: $28 - $88

cBPCR	Strategy	Cost (CAD)	Effectiveness	NHB (QALYs)
28	Don’t Treat Anyone	608.1947	14.99613	14.98396
28	Clinical Judgement	611.0217	15.01453	15.00231
28	RIDT	622.5233	15.01748	15.00503
28	Batch PCR - Wait	631.2986	15.02414	15.01152
28	DIA	618.9916	15.03379	15.02141
28	NAAT	636.7511	15.04043	15.02769
28	Batch PCR - Treat	631.187	15.04498	15.03236
28	Treat Everyone	630.0087	15.04698	15.03438

cBPCR	Strategy	Cost (CAD)	Effectiveness	NHB (QALYs)
88	Don’t Treat Anyone	608.1947	14.99613	14.98396
88	Clinical Judgement	611.0217	15.01453	15.00231
88	RIDT	622.5233	15.01748	15.00503
88	Batch PCR - Wait	691.2986	15.02414	15.01032
88	DIA	618.9916	15.03379	15.02141
88	NAAT	636.7511	15.04043	15.02769
88	Batch PCR - Treat	691.187	15.04498	15.03116
88	Treat Everyone	630.0087	15.04698	15.03438

CAD, Canadian dollars; DIA, digital immunoassay; NAAT, nucleic acid amplification test; PCR, polymerase chain reaction; QALY, quality-adjusted life year; RIDT, rapid influenza diagnostic test
