# Supplementary material for: Point-of-care diagnostic tests for influenza in the emergency department: A cost-effectiveness analysis in a high-risk population from a Canadian perspective
Source: PLoS One. 2020 Nov 16;15(11):e0242255. doi: 10.1371/journal.pone.0242255 (PMC7668582; doi:10.1371/journal.pone.0242255)
Supplement: S2 Table — (PDF) [file pone.0242255.s004.pdf]

S2 Table. Parameters for children population

Variable	Base-case value	Range	Range Type†	Source
**Diagnostic Tests**				
**Influenza A**				
Sensitivity, children				
RIDT	0.612	0.55 - 0.672	Full	Merckx 2017[1]
DIA	0.876	0.818 - 0.922	Full	Merckx 2017
NAAT	0.902	0.792 - 0.958	Full	Merckx 2017
Clinical Judgement	0.36	0.22 - 0.52	Full	Dugas 2015 [2]
Batch PCR	0.95	0.75 - 1	Plausible	Assumption (Merckx 2017)
Specificity, children				
RIDT	0.992	0.985 - 0.997	Full	Merckx 2017
DIA	0.981	0.964 - 0.991	Full	Merckx 2017
NAAT	0.99	0.968 - 0.998	Full	Merckx 2017
Clinical Judgement	0.36	0.22 - 0.52	Full	Dugas 2015 [2]
Batch PCR	0.95	0.75 - 1	Plausible	Assumption (Merckx 2017)
**Influenza B**				
Sensitivity, children				
RIDT	0.657	0.453 - 0.805	Full	Merckx 2017
DIA	0.825	0.712 - 0.902	Full	Merckx 2017
NAAT	0.959	0.829 - 0.992	Full	Merckx 2017
Clinical Judgement	0.36	0.22 - 0.52	Full	Dugas 2015 [2]
Batch PCR	0.95	0.75 - 1	Plausible	Assumption (Merckx 2017)
Specificity , children				
RIDT	0.996	0.992 - 0.998	Full	Merckx 2017
DIA	0.988	0.956 - 0.997	Full	Merckx 2017
NAAT	0.995	0.982 - 0.999	Full	Merckx 2017
Clinical Judgement	0.78	0.72 - 0.83	Full	Dugas 2015
Batch PCR	0.95	0.75 - 1	Plausible	Assumption (Merckx 2017)
**ILI and Influenza-Related Probabilities**				
Adverse events, Tx	0.081	0.061 - 0.101	Plausible	Santesso 2019 (Unpublished)
Adverse events, no Tx	0.047	0.035 – 0.059	Plausible	Santesso 2019 (Unpublished)
Mortality (ICU admitted, Early Tx)	0.138	0.1 - 0.17	Plausible	Muthuri 2014[3]
Mortality (ICU-admitted, Late Tx)	0.116	0.09 - 0.15	Plausible	Muthuri 2014
Mortality (ICU-admitted, No Tx)	0.217	0.16 - 0.27	Plausible	Muthuri 2014
Mortality (Non-ICU, Early Tx)	0.023	0.02 - 0.03	Plausible	Muthuri 2014
Mortality (Non-ICU, Late Tx)	0.034	0.03 - 0.04	Plausible	Muthuri 2014
Mortality (Non-ICU, No Tx)	0.034	0.03 - 0.04	Plausible	Muthuri 2014

† Plausible ranges are defined as full where lower and upper limits were directly reported from the data source, and "plausible" where uncertainty of key parameter was not reported in the literature and so ± 25% was used to create a plausible range for sensitivity analysis.

DIA, digital immunoassay; ICU, intensive care unit; ILI, influenza-like-illness; NAAT, nucleic acid amplification test; PCR, polymerase chain reaction; QALY, quality-adjusted life year; RIDT, rapid influenza diagnostic test; Tx, treatment; y, years of age

References

1. Merckx J, Wali R, Schiller I, et al. Diagnostic accuracy of novel and traditional rapid tests for influenza infection compared with reverse transcriptase polymerase chain reaction. Ann Intern Med 2017;167:395-409.
2. Dugas AF, Valsamakis A, Atreya MR, et al. Clinical diagnosis of influenza in the ED. Am J Emerg Med 2015;33:770-775.
3. Muthuri SG, Venkatesan S, Myles PR, et al. Effectiveness of neuraminidase inhibitors in reducing mortality in patients admitted to hospital with influenza A H1N1pdm09 virus infection: A meta-analysis of individual participant data. Lancet Respir Med 2014;2:395-404.
