# Supplementary material for: Point-of-care diagnostic tests for influenza in the emergency department: A cost-effectiveness analysis in a high-risk population from a Canadian perspective
Source: PLoS One. 2020 Nov 16;15(11):e0242255. doi: 10.1371/journal.pone.0242255 (PMC7668582; doi:10.1371/journal.pone.0242255)
Supplement: S3 Table — (PDF) [file pone.0242255.s005.pdf]

S3 Table. Scenario analysis NHB results at a cost-effectiveness threshold of $50,000/QALY

	**Scenarios**							
	**Base-case**	**Cost of AE = Cost of ED visit**	**Early Tx Effect = Late Tx Effect**	**Tx had no benefit (mortality, quality-of-life, hospitalization)**	**Children (5 years of age)**	**Worst case (sensitivity and specificity LL)**	**Best case (sensitivity and specificity UL)**	**Individuals testing negative receiving NAI therapy**
**Increasing NHB (Low to High) →**	Don’t Treat Anyone (14.9839)	Don’t Treat Anyone (14.9839)	Don’t Treat Anyone (14.984)	Batch PCR – Treat (14.9824)	Don't Treat Anyone (70.2897)	Don’t Treat Anyone (14.984)	Don’t Treat Anyone (14.984)	Don't Treat Anyone (14.9840)
	Clinical Judgement (15.0023)	Clinical Judgement (15.0021)	Clinical Judgement (14.9869)	Treat Everyone (14.9824)	Clinical Judgement (70.3111)	Clinical Judgement (14.995)	RIDT (15.0099)	Batch PCR – Wait (15.0110)
	RIDT (15.005)	RIDT (15.005)	RIDT (14.9873)	Batch PCR – Wait (14.9827)	Batch PCR - Wait (70.3192)	RIDT (15.0006)	Clinical Judgement (15.0107)	Clinical Judgement (15.0183)
	Batch PCR–Wait (15.0109)	Batch PCR - Wait (15.0108)	DIA (14.9901)	NAAT (14.9831)	RIDT (70.3265)	Batch PCR - Wait (15.0047)	Batch PCR – Wait (15.0125)	RIDT (14.0195)
	DIA (15.0214)	DIA (15.0213)	Batch PCR - Wait (14.9906)	DIA (14.9835)	DIA (70.3417)	DIA (15.0162)	DIA (15.0256)	DIA (15.0277)
	NAAT (15.0277)	NAAT (15.0276)	NAAT (14.9909)	RIDT (14.9835)	NAAT (70.3437)	NAAT (15.0187)	NAAT (15.0324)	NAAT (15.0306)
	Batch PCR - Treat (15.0318)	Batch PCR - Treat (15.0315)	Batch PCR - Treat (14.9911)	Clinical Judgement (14.9836)	Batch PCR - Treat (70.3455)	Batch PCR - Treat Available (15.0213)	Treat Everyone (15.0344)	Batch PCR - Treat (14.0318)
	**Treat Everyone (15.0344)**	**Treat Everyone (15.0337)**	**Treat Everyone (14.9916)**	**Don’t Treat Anyone (14.9840)**	**Treat Everyone (70.3485)**	**Treat Everyone (15.0344)**	**Batch PCR - Treat (15.0344)**	**Treat Everyone (15.0344)**

CET, cost-effectiveness threshold; DIA, digital immunoassay tests; ED, emergency department; LL, lower limit; NAAT, nucleic acid amplification test; NAI, neuraminidase inhibitors; NHB, Net health benefit; PCR, polymerase chain reaction; QALYs, quality-adjusted life years; RIDT, rapid influenza diagnostic tests; Tx, treatment; UL, upper limit
