# Supplementary figures and images for: Point-of-care diagnostic tests for influenza in the emergency department: A cost-effectiveness analysis in a high-risk population from a Canadian perspective
Source: PLoS One. 2020 Nov 16;15(11):e0242255. doi: 10.1371/journal.pone.0242255 (PMC7668582; doi:10.1371/journal.pone.0242255)

S1 Fig. Cost-effectiveness acceptability curve

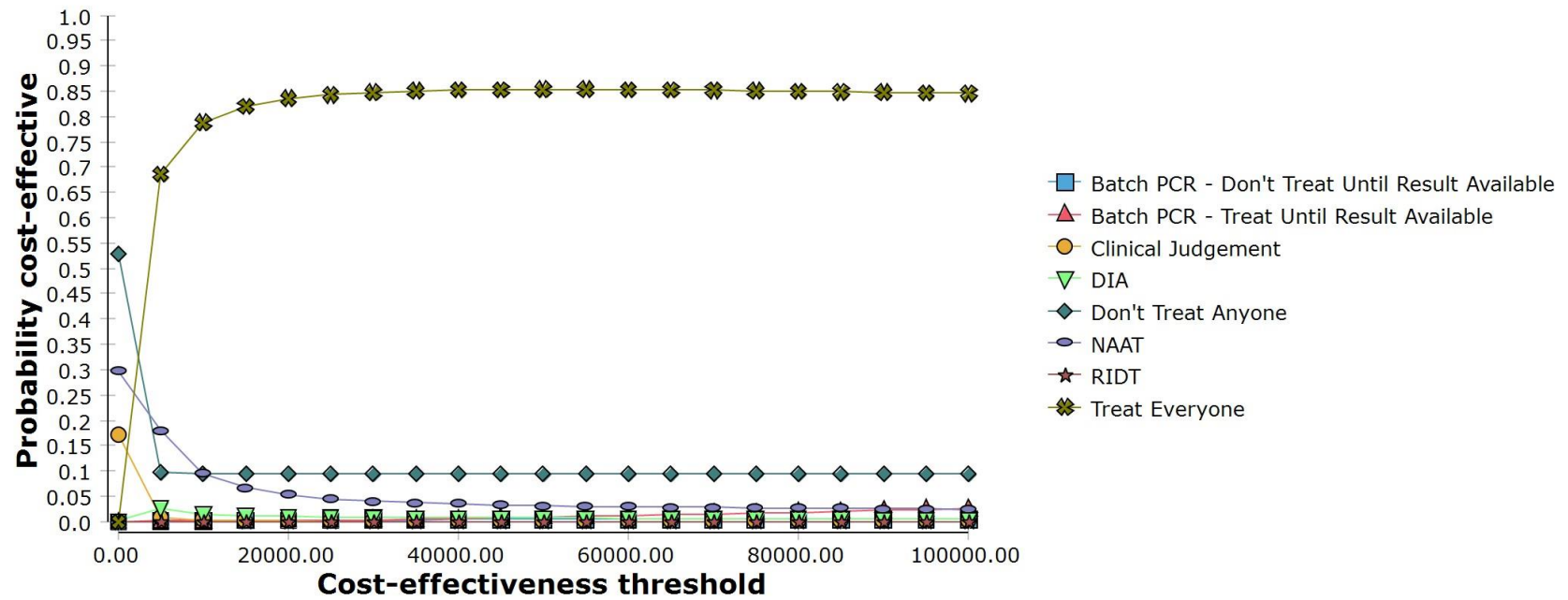

Supplement: S1 Fig — (PDF) [file pone.0242255.s006.pdf]
