# Supplementary material for: Point-of-care diagnostic tests for influenza in the emergency department: A cost-effectiveness analysis in a high-risk population from a Canadian perspective
Source: PLoS One. 2020 Nov 16;15(11):e0242255. doi: 10.1371/journal.pone.0242255 (PMC7668582; doi:10.1371/journal.pone.0242255)
Supplement: Attachment — Submitted filename: PONE-D-20-15378 R1 Ebell final comments.docx [file pone.0242255.s008.docx]

1. The authors write:

Other meta-analysis in the literature have suggested no evidence of mortality benefit from oseltamivir[26]. However, since this review includes RCTs of low-risk patients or mixed populations (i.e., the large majority of enrolled patients did not have severe influenza infection), the evidence is highly indirect for our study’s target population.

I suggest revising this as follows and adding reference to the Cochrane Review by Jefferson and colleagues.

Meta-analyses of randomized controlled trials have suggested no evidence of mortality benefit from oseltamivir[26,<INSERT COCHRANE REFERENCE>]. However, since this review includes RCTs of low-risk patients or mixed populations (i.e., the large majority of enrolled patients did not have severe influenza infection), the evidence is highly indirect for our study’s target population.

2. There should be a clear statement to the effect that these results apply only to high risk elderly patients being evaluated in the ED setting and should not be extrapolated to lower risk populations or to other settings such as primary care, where risk of mortality and hospitalization and cost of care are all much lower, but the adverse events related to the drug and cost of the drug remain the same.

3. There should also be a discussion of the magnitude of the difference in QALYs, costs, and net health benefit between strategies. For example, in Table 2 the QALYs for NAAT strategy were 15.0404 and for treat everyone were 15.0470. This is a gain of only 0.0066 QALYs or 2.4 days on average, which is quite small.
