# Supplementary material for: Point-of-care diagnostic tests for influenza in the emergency department: A cost-effectiveness analysis in a high-risk population from a Canadian perspective
Source: PLoS One. 2020 Nov 16;15(11):e0242255. doi: 10.1371/journal.pone.0242255 (PMC7668582; doi:10.1371/journal.pone.0242255)
Supplement: Attachment — Submitted filename: Response to Reviewers.docx [file pone.0242255.s009.docx]

Stephen Mac

Institute of Health Policy, Management, and Evaluation

University of Toronto

155 College St. Suite 425

Toronto, ON M5T3M6

Email: sm.mac@mail.utoronto.ca

To: Ruslan Kalendar

Academic Editor

PLOS ONE

October 28, 2020

**RE: PONE-D-20-15378-R1 “Point-of-Care Diagnostic Tests for Influenza in the Emergency Department: A Cost-Effectiveness Analysis in a High-Risk Population from a Canadian Perspective”**

Dear Dr. Kalendar,

Thank you for taking the time to review our manuscript and providing additional comments. We have addressed all comments and suggestions and revised our manuscript accordingly for resubmission to *PLOS One.* Please find enclosed our revised manuscript entitled “Point-of-Care Diagnostic Tests for Influenza in the Emergency Department: A Cost-Effectiveness Analysis in a High-Risk Population from a Canadian Perspective”.

Following this letter, we included a table outlining our detailed response to all comments for your review. Thank you for your time and consideration. We look forward to your final decision.

Sincerely,

Stephen Mac

*On behalf of all authors below*

**Stephen Mac *PhD(c), MBiotech***

Institute of Health Policy, Management and Evaluation, University of Toronto, Toronto, Canada

Toronto Health Economics and Technology Assessment (THETA) Collaborative, University Health Network, Toronto, Canada

**Ryan O’Reilly *MD(c), PhD(c)***

Department of Medicine, McMaster University, Hamilton, Canada

Institute of Health Policy, Management and Evaluation, University of Toronto, Toronto, Canada

Toronto Health Economics and Technology Assessment (THETA) Collaborative, University Health Network, Toronto, Canada

**Neill Adhikari *MDCM, MSc***

Department of Critical Care Medicine, Sunnybrook Health Sciences Centre, Toronto, Canada

Interdepartmental Division of Critical Care Medicine, University of Toronto, Toronto, Canada

**Rob Fowler *MDCM, FRCPC, MSc***

Institute of Health Policy, Management and Evaluation, University of Toronto, Toronto, Canada

Department of Critical Care Medicine, Sunnybrook Health Sciences Centre, Toronto, Canada

Interdepartmental Division of Critical Care Medicine, University of Toronto, Toronto, Canada

**Beate Sander *PhD***

Institute of Health Policy, Management and Evaluation, University of Toronto, Toronto, Canada

Toronto Health Economics and Technology Assessment (THETA) Collaborative, University Health Network, Toronto, Canada

Public Health Ontario, Toronto, Canada

ICES, Toronto, Canada

| **Reviewer** | **Response** |
| --- | --- |
| The authors write: Other meta-analysis in the literature have suggested no evidence of mortality benefit from oseltamivir[26]. However, since this review includes RCTs of low-risk patients or mixed populations (i.e., the large majority of enrolled patients did not have severe influenza infection), the evidence is highly indirect for our study’s target population.  I suggest revising this as follows and adding reference to the Cochrane Review by Jefferson and colleagues.  Meta-analyses of randomized controlled trials have suggested no evidence of mortality benefit from oseltamivir[26,]. However, since this review includes RCTs of low-risk patients or mixed populations (i.e., the large majority of enrolled patients did not have severe influenza infection), the evidence is highly indirect for our study’s target population. | We have revised as suggested on page 9 (line 170 to 172). |
| There should be a clear statement to the effect that these results apply only to high risk elderly patients being evaluated in the ED setting and should not be extrapolated to lower risk populations or to other settings such as primary care, where risk of mortality and hospitalization and cost of care are all much lower, but the adverse events related to the drug and cost of the drug remain the same. | We have revised as suggested in the limitations of the Discussion on page 19 (line 346 to 349). |
| There should also be a discussion of the magnitude of the difference in QALYs, costs, and net health benefit between strategies. For example, in Table 2 the QALYs for NAAT strategy were 15.0404 and for treat everyone were 15.0470. This is a gain of only 0.0066 QALYs or 2.4 days on average, which is quite small. | We have revised as suggested in the first paragraph of the Discussion on page 17 (line 311 to 314). |
